# The prognostic importance of the pan-immune-inflammation value in patients with septic shock

**DOI:** 10.1186/s12879-023-08963-w

**Published:** 2024-01-10

**Authors:** Yasemin Bozkurt Turan

**Affiliations:** Department of Critical Care, Marmara University Faculty of Medicine, Pendik, Istanbul, 34899 Turkey

**Keywords:** Pan-immune-inflammation value, Septic shock, Intensive care

## Abstract

**Introduction:**

The purpose of this study was to determine whether the pan-immune-inflammation value (PIV), a novel biomarker combining neutrophil platelet, monocyte, and lymphocyte counts, some of the most widespread indicators of systemic inflammation, can predict mortality and prognosis in patients admitted to the intensive care unit (ICU) with septic shock.

**Method:**

This prospective study was performed with 82 patients aged 18 or over admitted to a tertiary ICU with diagnoses of septic shock. Patients with hematological disease and neutropenia were excluded. PIV was calculated with the formula [neutrophil count (10^3^/μL) × platelet count (10^3^/μL) × monocyte count (10^3^/μL)]/lymphocyte count (10^3^/μL).

**Results:**

Median age, presence of hypertension, Acute Physiology and Chronic Health Evaluation II (APACHE II) levels, and neutrophil, monocyte, and platelet counts were lower in the low-PIV group than in the high-PIV group (*p* < 0.05). The highest area under ROC curve (AUC) was determined for Sequential Organ Failure Assessment (SOFA) (0.94 (0.89 – 0.99)), followed by Glasgow Coma Scale (GCS) (0.81 (0.70 – 0.91)), APACHE II (0.80 (0.69 – 0.91)) and lactate (0.77 (0.67 – 0.88)). Median survival was longer in the low-PIV group than in the high-PIV group (28 (15.25 – 40.76) vs 16 (9.46 – 22.55) days, respectively, *p* < 0.05). The univariate Cox proportional hazards (CPH) model showed that high PIV (HR = 2.13 (1.03—4.38)), low GCS (HR = 3.31 (1.34 – 8.15)), high SOFA (HR = 9.41 (2.86 – 30.95)), high APACHE II (HR = 3.08 (1.47 – 6.45)), high lactate (HR = 6.56 (2.73 – 15.75)), and high procalcitonin (PCT) (HR = 2.73 (1.11 – 6.69)) values were associated with a decreased survival time among ICU patients (*p* < 0.05). The multivariate CPH model showed the age-adjusted risk estimates for these six laboratory parameters. High lactate (HR = 7.97 (2.19 – 29.08)) and high SOFA scores (HR = 4.85 (1.22 – 19.32)) were significantly associated with shorter survival in ICU patients (*p* < 0.05).

**Conclusion:**

The findings of this research suggest that PIV could predict the longer survival in patients with septic shock. Despite PIV score’s capability to show inflammation, it is not significantly associated with mortality in the multivariate analysis.

## Introduction and objective

Sepsis is a life-threatening organ dysfunction deriving from a dysregulated host response to infection [1]. The incidence of sepsis and septic shock have risen continually since the first consensus definition in 1991, reaching approximately 49 million cases worldwide in 2017, with 11 million sepsis-related deaths. These data led to sepsis being declared a global health priority by the World Health Organization (WHO) [2]. Early diagnosis and appropriate treatment in the first hours following the development of sepsis and septic shock improve outcomes [1]. Testing other methods of showing systemic inflammation for identifying high-risk septic shock patients may therefore be a useful guide. From the pathogenetic perspective, sepsis is currently regarded as the result of several mechanisms involving numerous pro- and anti-inflammatory mediators on a simultaneous basis [3]. The pan-immune-inflammation value (PIV) [neutrophil count (10^3^/mm^3^) × platelet count (10^3^/mm^3^) × monocyte count (10^3^/mm^3^)]/lymphocyte count (10^3^/mm^3^), a novel biomarker that combines neutrophil, platelet, monocyte, and lymphocyte counts, some of the most widespread indicators of systemic inflammation, is an index capable of evaluating patients’ immune and inflammatory status [4, 5]. PIV has recently begun being employed as an inflammatory markers for various. Studies have shown that a high PIV indicates poor prognosis in cancer patients [6], is a reliable predictor of clinical outcomes in cancer patients [7], constitutes a new prognostic biomarker in patients with metastatic colorectal cancer [8, 9], and is associated with increased mortality in hypertensive patients [5].

To the best of our knowledge, no previous studies have investigated the relationship between PIV and mortality and prognosis in patients with septic shock. The purpose of this study was to determine whether PIV can predict mortality and prognosis in patients admitted to the intensive care unit (ICU) with septic shock. In addition, PIV was compared with the Sequential Organ Failure Assessment (SOFA) score, the Acute Physiology and Chronic Health Evaluation II (APACHE II) score, lactate, C-reactive protein (CRP), and procalcitonin (PCT), routinely employed in clinical practice, to determine which may best predict mortality in septic shock.

## Methods

### Patients

**Fig. 1:**
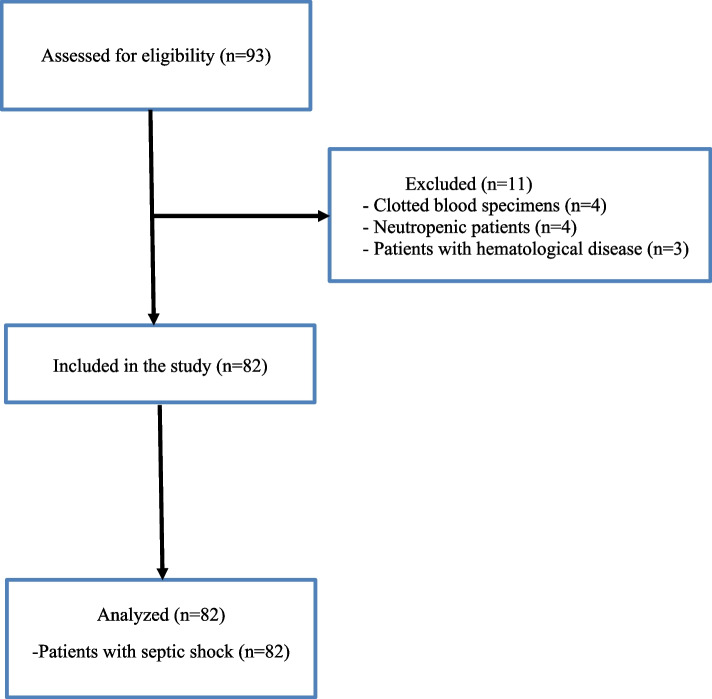
Study design flowchart

This prospective study was performed in patients aged 18 or over who were diagnosed with septic shock while being followed up in the tertiary ICU of Marmara University Hospital between July 2022 and January 2023, and in whom informed and written consent was obtained from themselves or their relatives. The research was conducted in conformity with the Declaration of Helsinki and Best Clinical Practice guidelines. Approval was granted by the Marmara University Medical Faculty clinical research ethical committee (no: 09.2022.1023). The complete blood count (CBC) was obtained in patients who developed septic shock while hospitalised in the ICU. Diagnosis of septic shock was based on Surviving Sepsis Campaign criteria [1]. Patients with hematological diseases and neutropenia were excluded. CBC was performed to evaluate the patient’s neutrophil, platelet, monocyte, and lymphocyte counts. PIV was calculated from those data. For CBC measurement, blood specimens collected from the catheter in the patient’s radial artery were placed into ethylene diamine tetra-acetic acid (EDTA)-containing tubes. The washing solution was removed from the arterial catheter before blood sampling. Ninety-three patients were admitted to intensive care during the study period. However, clotting occurred in four blood specimens, four patients were neutropenic, and three patients had hematological diseases. These were excluded from the study, which was thus completed with 82 patients with septic shock (Fig. 1).

The Strengthening the Reporting of Observational Studies in Epidemiology (STROBE) case–control checklist was used in the writing of this article [10]. Age, sex, BMI, comorbid diseases, lactate, neutrophil, monocyte, platelet, lymphocyte, PIV, CRP, and procalcitonin values, Glasgow Coma Scale (GCS) and sequential organ failure assessment (SOFA) scores, and Acute Physiology and Chronic Health Evaluation II (APACHE II) values were recorded at the time of diagnosis of septic shock. PIV was calculated using the formula [neutrophil count (10^3^/μL) × platelet count (10^3^/μL) × monocyte count (10^3^/μL)]/lymphocyte count (10^3^/μL) [11]. Whether a correlation existed between PIV values and mortality was investigated. The patients were followed-up at least 28 days from admission to the ICU.

### Sample size

The sample size estimated based on high- and low-PIV groups’ comparison of survivor functions. The median PIV value used as the cut-off value to form the groups, and the patients were allocated on 1:1. The estimated total sample size was 74 patients when the high- to low-PIV groups had hazard ratio (HR) of 2.0 based on previous studies on different fields [6, 11–14] along with alpha level of 0.05 and power of 80% (Stata Statistical Software: Release 15. College Station, TX: Stata Corp LLC).

### Statistical analysis

Descriptive statistics were presented as frequency (%) for categorical variables, and as median (IQR) and mean (± SD) for continuous variables. Normal distribution assumptions were assessed using the Shapiro–Wilk test. The median PIV value was adopted as the cut-off between low and high PIV. Patient characteristics were compared between the low- and high-PIV groups using the Mann–Whitney U test and Pearson’s chi-square test. Cut-off values of the laboratory parameters predicting ICU mortality were determined using receiver operating characteristics (ROC) curve analysis and the maximum Youden Index value. Overall survival (OS) time and survival probability were estimated with the Kaplan–Meier method. The survival curves of the low- and-high PIV groups were compared using the Wilcoxon (Breslow) test. The clinical and laboratory parameters predicting ICU survival time were examined with the multivariate Cox proportional hazards (CPH) model, enter method. Schoenfeld’s residuals were investigated for proportional hazards assumption. Statistical significance was set at 0.05 level. Statistical analysis was performed on Jamovi version 2.3 software (The Jamovi project (2022) and R Core Team (2021)), and STATA 15 (Stata Statistical Software: Release 15. College Station, TX, USA: Stata Corp LLC.) for testing the proportional hazards assumption.

## Results

### Patient characteristics

The clinical and laboratory parameters of the 82 ICU patients are presented in Table 1. The patients’ mean age was 62.63 (± 18.10) years, and 52.44% were male. Mean BMI was 28.86 (± 12.08). Twelve (14.63% patients were diagnosed with malignancy, 23 (28.05%) with respiratory diseases, 10 with (12.20%) operational diseases, and 37 (45.12%) with other diseases. Comorbidity was present in 58 (70.73%) patients. Hypertension (HT) was present in 35 (42.68%) patients, diabetes mellitus (DM) in 18 (21.95%), and cardiovascular disease (CVD) in 17 (20.73%) patients. Mean and median GCS, SOFA, and APACHE II, and lactate values, neutrophil, monocyte, platelet, and lymphocyte counts, and PIV, CRP and PCT values are presented in Table 1. The mortality rate was 39.02% (*n* = 32).

**Table 1:** Clinical and laboratory parameters

**Parameter**	n	Median (IQR)
Age	82	64.50 (51.00 – 77.00)
Sex (M/F)	43/39	
BMI kg/m^2^	75	27.13 (23.44 – 30.86)
Diagnosis, n (%)		
Malignity	12 (14.63)	
Respiratory	23 (28.05)	
Operational	10 (12.20)	
Other	37 (45.12)	
Comorbidity presence, n (%)		
Yes	58 (70.73)	
No	24 (29.27)	
HT, n (%)	35 (42.68)	
DM, n (%)	18 (21.95)	
CVD, n (%)	17 (20.73)	
Presence of other diseases, n (%)	27 (32.93)	
GCS	82	13.00 (3.00 – 15.00)
SOFA	82	7.00 (2.00 – 12.00)
APACHE II	81	17.00 (9.00 – 24.00)
Lactate mmol/L	82	1.80 (1.10 – 3.40)
Neutrophil × 10^3^/μL	82	10.00 (7.30 – 13.20)
Monocyte × 10^3^/μL	82	0.50 (0.40 – 0.90)
Platelet × 10^3^/μL	82	192.50 (127.00 – 244.00)
Lymphocyte × 10^3^/μL	82	0.90 (0.60 – 1.60)
PIV × 10^6^/μL	82	1174.17 (489.70 – 2341.73)
CRP mg/L	82	94.74 (40.2–166)
PCT µg/L	82	0.72 (0.24 – 5.27)
Mortality, n (%)	32 (39.02)	

### Clinical and laboratory parameters of the Low- and High-PIV Groups

Clinical and laboratory parameters were compared between the low- and high-PIV groups (Table 2 and 3). Median age, presence of HT, and APACHE II, neutrophil, monocyte and platelet values were lower in the low-PIV group than in the high-PIV group (*p* < 0.05).

**Table 2:** Clinical and laboratory parameters of the Low- and High-PIV Groups

**Parameter**	**Low PIV (*n* = 41)**	**High PIV (*n* = 41)**	p; Test statistic
Age^a^	63.00 (51.00–67.00)	74.00 (59.00–81.00)	***p* = 0.010; W = 563.00**^**1**^
Sex (M/F)	20/21	23/18	*p* = 0.507; ${\chi }^{2}$=0.44^2^
BMI^ab^	24.42 (22.96–30.81)	27.34 (24.06–30.86)	*p* = 0.119; W = 554.50^1^
Diagnosis, n (%)			*p* = 0.392; ${\chi }^{2}$=2.30^2^
Malignity	7 (58.33%)	5 (41.67%)	
Respiratory	10 (43.48%)	13 (56.52%)	
Operational	3 (30.00%)	7 (70.00%)	
Other	21 (56.76%)	16 (43.24%)	
Comorbidity, n (%)			*p* = 0.145; ${\chi }^{2}$=2.12^2^
Present	26 (44.83%)	32 (55.17%)	
not present	15 (62.50%)	9 (37.50%)	
HT, n (%)	10 (28.57%)	25 (71.43%)	***p* < 0.001; **${{\boldsymbol{\chi}}}^{2}$**=11.22**
DM, n (%)	7 (38.89%)	11 (61.11%)	*p* = 0.286; ${\chi }^{2}$=1.14^2^
CV, n (%)	6 (35.29%)	11 (64.71%)	p = 0.173; ${\chi }^{2}$=1.86^2^
Other disease, n (%)	18 (66.67%)	9 (33.33%)	***p* = 0.034; **${{\boldsymbol{\chi}}}^{2}$**=4.47**^2^
GCS^a^	14.00 (6.00–15.00)	8.00 (3.00–15.00)	*p* = 0.126; W = 998.50^1^
SOFA^a^	6.00 (1.00–10.00)	9.00 (2.00–12.00)	*p* = 0.173; W = 693.50^1^
APACHE II^ac^	13.00 (7.50–20.50)	21.00 (14.00–28.00)	***p* = 0.015; W = 561.50**^**1**^
Lactate mmol/L^a^	1.90 (1.30–3.00)	1.70 (1.10–3.80)	*p* = 0.937; W = 831.50^1^
CRP mg/L^a^	94.2 (29.7–143)	98.00 (48.50–180.00)	*p* = 0.266; W = 720.00^1^
PCT µg/L^a^	0.65 (0.31–4.34)	0.89 (0.24–5.80)	*p* = 0.492; W = 766.00^1^

^a^Descriptive statistics reported as median (IQR)

^b^Comparison between Low PIV *n* = 36 vs High PIV *n* = 39

^c^comparison between Low PIV *n* = 40 vs High PIV *n* = 41

^1^Mann-Whitney U test statistic

^2^Pearson Chi-square test statistic

**Table 3:** CBC Counts of the Low- and High-PIV Groups

**Parameter**^**a**^	**Low PIV (*n* = 41)**	**High PIV (*n* = 41)**	p; Test statistic
Neutrophil	7.80 (5.50–10.10)	12.40 (8.80–15.60)	***p* = < 0.001; W = 320.50**^**1**^
Monocyte	0.50 (0.30–0.70)	0.70 (0.50–1.10)	***p* = < 0.001; W = 464.00**^**1**^
Platelet	149.00 (84.00–205.00)	228.00 (178.00–267.00)	***p* = < 0.001; W = 474.00**^**1**^
Lymphocyte	1 (0.60–1.70)	0.80 (0.60–1.40)	*p* = 0.227; W = 971.00^1^
PIV × 10^6^/μL	489.7 (323.85–966.43)	2341.73 (1678.29–3079.27)	***p* = < 0.001; W = 0.00**^**1**^

^a^Descriptive statistics reported as median (IQR)

^1^Mann-Whitney U test statistic

### Cut-off values of the laboratory parameters

The cut-off values of the laboratory parameters predicting ICU mortality and the test performance indicators are presented in Table 4. The highest area under the ROC curve (AUC) values were estimated for SOFA (0.94 (0.89 – 0.99)), followed by GCS (0.81 (0.70 – 0.91)), APACHE II (0.80 (0.69 – 0.91)) and lactate (0.77 (0.67 – 0.88)). The AUC values for CRP (0.64 (0.52 – 0.76)) and PCT (0.67 (0.55 – 0.79)) were lower than 0.70. The classification of the mortality status was not statistically significant for PIV (AUC (95% CI) = 0.58 (0.44 – 0.72); *p* = 0.227).

**Table 4:** Cut-off values for the laboratory parameters

Parameter	Cut-off value^a^	Sensitivity	Specifity	AUC (95% CI)
GCS	9.00	81.30%	78.00%	0.81 (0.70 – 0.91)
SOFA	8.50	90.60%	88.00%	0.94 (0.89 – 0.99)
APACHE II	22.50	64.50%	90.00%	0.80 (0.69 – 0.91)
Lactate mmol/L	2.05	78.10%	74.00%	0.77 (0.67 – 0.88)
CRP	102.29	62.50%	66.00%	0.64 (0.52 – 0.76)
PCT µg/L	0.63	81.30%	58.00%	0.67 (0.55 – 0.79)
PIV	1777.02	50.00%	78.00%	0.58 (0.44 – 0.72)

^a^Assessed with maximum Youden Index value for the ROC analysis predicting ICU mortality

### Survival analysis

**Fig. 2:**
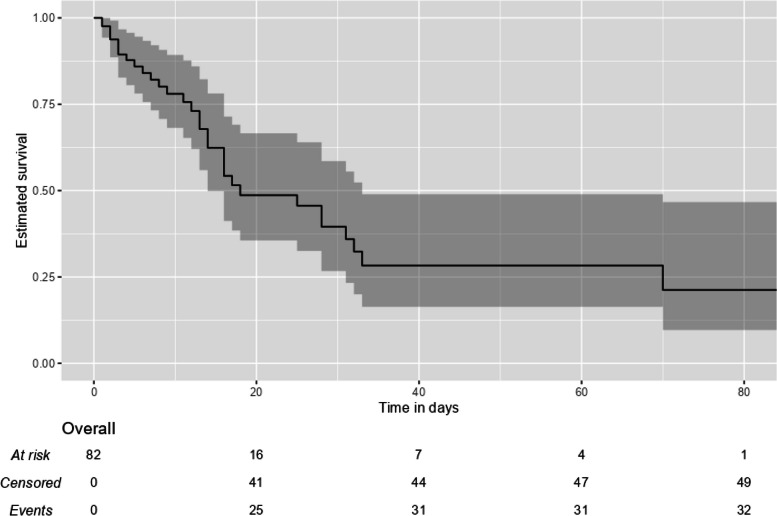
Kaplan–Meier Survival Curve Estimate for Overall Survival in the ICU

**Fig. 3:**
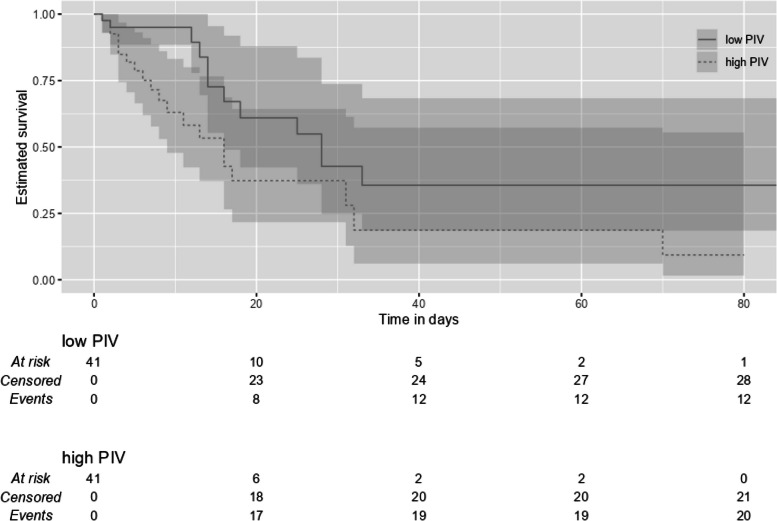
Kaplan–Meier Survival Curves of Overall Survival in the Low- and High-PIV Groups

The OS values of the ICU patients are shown in Table 5 The OS rate among all the ICU patients was 61%, and the median survival time was 18 (8.65 – 27.35) days. Kaplan–Meier survival curves for all the ICU patients are shown in Fig. 2. Median survival was longer in the low-PIV group than in the high-PIV group (28 (15.25 – 40.76) vs 16 (9.46 – 22.55) days, respectively, *p* < 0.05). The survival probability for 28-day was 39.56% in all patients, while it was 42.70% and 37.30% in low- and high-PIV groups, respectively (Table 5). Kaplan–Meier survival curves for the low- and high-PIV groups are shown in Fig. 3.

**Table 5:** Overall Survival of the ICU patients

	Survival estimate (95% CI)	N of events (%)	Breslow test p
OS (days)^a^			
All patients	18.00 (8.65 – 27.35)	32 (39.02)	
Low-PIV	28.00 (15.25 – 40.76)	12 (29.30)	Chi-square = 4.776
High-PIV	16.00 (9.46 – 22.55)	20 (48.80)	***p* = 0.029**^**b**^
28-day OS probability^c^			
All patients	39.60% (24.10% – 55.00%)	28 (34.15)	
Low-PIV	42.70% (24.70%—73.70%)	11 (26.83)	
High-PIV	37.30% (21.70%—64.30%)	17 (41.46)	

Kaplan–Meier estimate of ^a^median survival time and ^c^survival probability. ^b^Breslow test *p*-value comparing survivor functions between low- and high- PIV groups

*OS* overall survival

Clinical and laboratory parameters associated with ICU mortality in the septic shock patients were investigated with the univariate CPH model presented in Table 6. High PIV (HR = 2.13 (1.03—4.38)), low GCS (HR = 3.31 (1.34 – 8.15)), high SOFA (HR = 9.41 (2.86 – 30.95)), high APACHE II (HR = 3.08 (1.47 – 6.45)), high lactate (HR = 6.56 (2.73 – 15.75)), and high PCT (HR = 2.73 (1.11 – 6.69)) were associated with decreased survival time (*p* < 0.05). In the multivariate model showed the age-adjusted risk estimates for these six laboratory parameters. High lactate (HR = 7.97 (2.19 – 29.08)) and high SOFA (HR = 4.85 (1.22 – 19.32)) were significantly associated with declined survival time (*p* < 0.05). Patients with high lactate levels (≥ 2.05) had 7.97 times higher risk of mortality than those with low lactate levels. Similarly, patients with high SOFA scores (≥ 8.50) had 4.85 times higher risk of mortality than those with low SOFA scores (Table 6).

**Table 6:** Cox Proportional Hazard Model Predicting ICU Mortality

Covariate (ref.)	Univariate		Multivariate^a^	
	HR (95% CI)	p	HR (95% CI)	p
Age ≥ 65 (< 65)	1.83 (0.88 – 3.80)	0.107	0.61 (0.24 – 1.56)	0.298
Male (Female)	1.39 (0.68 – 2.86)	0.365		
BMI < 30 (≥ 30)	1.63 (0.69 – 3.84)	0.265		
Diagnosis (Other)		0.449		
Malignity	1.32 (0.41 – 4.31)	0.642		
Respiratory	1.92 (0.86 – 4.29)	0.110		
Operational	0.01 (0.01 – 520.16)	0.968		
Comorbidity absence (presence)	1.38 (0.65 – 2.94)	0.405		
HT presence (absence)	0.96 (0.48 – 1.94)	0.914		
DM presence (absence)	1.07 (0.46 – 2.52)	0.873		
CV presence (absence)	1.01 (0.44 – 2.31)	0.981		
High PIV (Low PIV)	2.13 (1.03 – 4.38)	**0.041**	1.77 (0.73 – 4.26)	0.204
Low GCS (High GCS)	3.31 (1.34 – 8.15)	**0.009**	2.18 (0.78 – 6.10)	0.136
High SOFA (Low SOFA)	9.41 (2.86 – 30.95)	** < 0.001**	4.85 (1.22 – 19.32)	**0.025**
High APACHE II (Low APACHE II)	3.08 (1.47 – 6.45)	**0.003**	0.86 (0.33 – 2.28)	0.762
High Lactate mmol/L (Low Lactate)	6.56 (2.73 – 15.75)	** < 0.001**	7.97 (2.19 – 29.08)	**0.002**
High CRP mg/L (Low CRP)	1.49 (0.72 – 3.08)	0.278		
High PCT µg/L (Low PRT)	2.73 (1.11 – 6.69)	**0.029**	0.41 (0.12 – 1.42)	0.160

*HR* Hazard Ratio

^a^Proportional Hazards assumption tested with Schoenfeld residuals (*p* > 0,05 satisfied for each parameter)

### Discussion

Sepsis and septic shock are important health problems that affect millions across the world every year and that result in mortality in one in three and one in six, respectively, of those affected [1]. In the face of this significant risk of mortality the screening of patients with septic shock and early intervention are of critical importance. New diagnostic methods for identifying patients with septic shock may therefore need to be tested. The PIV, one of these novel methods, began employed as an inflammatory marker in recent years. The PIV is particularly advantageous as a low-cost, simple, and easily available parameter obtained from complete blood count tests CBC. Research has shown that sepsis and septic shock are an immune and inflammatory disease [15]. The PIV is an index that combines neutrophils, platelets. Monocytes, and lymphocytes, and has been regarded as representing a comprehensive evaluation of immune and inflammatory conditions in previous studies evaluation [5, 9]. Thrombocytosis emerges as the result of the stimulation of megakaryocytes by proinflammatory cytokines [16, 17]. Platelets are not solely associated with thrombosis, but also trigger and exacerbate inflammation by combining with endothelial cells and encouraging leukocyte migration and adhesion. Monocytes reflect the function of macrophages, and the numbers of these proinflammatory cells generally indicate the patient’s immune and inflammatory status [18, 19]. Since the reactions of leukocytes in circulation to various inflammatory events is generally characterized by an increase in neutrophil numbers and a decrease in lymphocytes, the ratio between them is employed as an inflammatory marker in the clinical setting in the ICU [20, 21]. The PIV appears to be a powerful indicator of mortality in patients diagnosed with ST-segment elevation myocardial infarction (STEMI) examined retrospectively [5, 22]. A study of patients with local advanced head and neck squamous cell carcinoma (HNSCC) observed shorter overall survival (OS) and disease-free survival in patients with high PIV values. The results suggested that PIV scores may represent a prognostic biomarker in HNSCC [23]. A meta-analysis involving patients with malignant tumors showed that patients with higher PIV values were at a significantly higher risk of mortality than those with low PIV scores (HR = 2.00, 95% CI: 1.51–2.64, *p* < 0.001) [6]. Patients with advanced triple-negative breast cancer (aTNBC) with high PIV values experienced poorer OS [adjusted hazard ratio (HR): 4.46, 95% confidence interval (CI): 2.22–8.99; adjusted *p* < 0.001] [12]. In a retrospective study of breast cancer patients with a PIV cut-off value of 310.2 [24], five year OS rates in the low- and high-PIV groups were 71.55% and 62.50%, respectively (hazard ratio (HR): 1.737, 95% CI: 1.096–2.755, log-rank test, *p* = 0.016) [5]. At multivariate Cox hazard model analysis of patients diagnosed with antineutrophil cytoplasmic antibody (ANCA)-associated vasculitis (AAV) [13] whose medical records were investigated PIV ≥ 1011.3 (HR 2.689, 95% CI 1.156, 6.254) emerged as a significant and independent risk factor for all-cause mortality. PIV predicted mortality in these studies, the majority of which were conducted retrospectively.

In this prospective study, we aimed to examine the role of PIV for predicting the mortality and prognosis in ICU patients with septic shock. The median PIV value used for formation of the low- and high-PIV groups, and clinical and laboratory parameters of eighty-two patients were compared between the groups. The median age, presence of HT, APACHE II score, and neutrophil, monocyte, and platelet counts were significantly lower in the low-PIV group than the high-PIV group. This finding is in accordance with the literature except for APACHE II score [5]. There was no significant difference observed in lymphocyte counts. The similar lymphocyte level between low- and high-PIV groups may be related with PIV’s inability to predict the mortality in multivariate analysis. The prognostic PIV value estimated with ROC analysis showed that the AUC value was insignificant, thus it did not provide sufficient evidence to classify the mortality. When the survival analysis rerun with the prognostic value, it showed similar results with the median PIV value findings presented in this study. The median survival time was significantly higher in the low-PIV group than the high-PIV group, this finding was in accordance with the few studies [23–26]. The univariate Cox PH model showed that the high PIV value was a risk factor in survival, which was similar to those of previous studies [24, 26].

The multivariate analysis indicated that the lactate elevation and high SOFA scores associated with risk factors of survival, while PIV was an insignificant risk factor. The insignificance of high-PIV as a risk factor might be due to the confounding effect of age and other inflammatory factors that were included in the multivariate analysis, since these parameters were correlated with each other, and high PIV was also correlated with elevated APACHEE II level and older age. The insufficient sample size in the multivariate analysis could be another factor contributing to lack of evidence in demonstrating the association of PIV and mortality. According to our knowledge, this is the first study investigating PIV in the septic shock patients.

## Conclusion

In conclusion, our finding showed that PIV could significantly predict the median survival time difference in patients with septic shock. Although PIV is capable of showing inflammation, it is not associated with mortality in the multivariate analysis. Further studies are needed to better understand the relationship between PIV and mortality in the septic shock patients.

## Data Availability

The datasets used and/or analysed during the current study available from the corresponding author on reasonable request.

## Abbreviations

PIV: Pan-immune-inflammation value
ICU: Intensive care unit
APACHE II: Acute Physiology and Chronic Health Evaluation II
AUC: Area under ROC curve
SOFA: Sequential Organ Failure Assessment
GCS: Glasgow Coma Scale
CPH: Cox proportional hazards
PCT: Procalcitonin
CRP: C-reactive protein
WHO: World Health Organization
CBC: Complete blood count
EDTA: Ethylene diamine tetra-acetic acid
STROBE: Strengthening the Reporting of Observational Studies in Epidemiology
ROC: Receiver operating characteristics
OS: Overall survival
CPH: Cox proportional hazards
HT: Hypertension
DM: Diabetes mellitus
CVD: Cardiovascular disease
AUC: Area under curve
STEMI: ST-segment elevation myocardial infarction
HNSCC: Head and neck squamous cell carcinoma
OS: Overall survival
ANCA-AAV: Antineutrophil cytoplasmic antibody-Associated vasculitis

## References

[1] Evans L, Rhodes A, Alhazzani W, Antonelli M, Coopersmith CM, French C, et al. Surviving sepsis campaign: International guidelines for management of sepsis and septic shock 2021. Intensive Care Med. 2021;47:1181–247. 34599691 10.1007/s00134-021-06506-y PMC8486643

[2] Guarino M, Perna B, Cesaro AE, Maritati M, Spampinato MD, Contini C, et al. 2023 Update on Sepsis and Septic Shock in Adult Patients: Management in the Emergency Department. J Clin Med. 2023;12(9):3188. 37176628 10.3390/jcm12093188 PMC10179263

[3] Piechota M, Banach M, Irzmanski R, Barylski M, Piechota-Urbanska M, Kowalski J, et al. Plasma endothelin-1 levels in septic patients. J Intensive Care Med. 2007;22:232–9. 17722367 10.1177/0885066607301444

[4] Yang XC, Liu H, Liu DC, Tong C, Liang XW, Hui R. Prognostic value of pan-immune-inflammation value in colorectal cancer patients: A systematic review and meta-analysis. Front Oncol. 2022;12:1036890 Published online 2022 Dec 22. 36620576 10.3389/fonc.2022.1036890 PMC9813847

[5] Wu B, Zhang C, Lin S, Zhang Y, Ding S, Song W. The relationship between the pan-immune-inflammation value and long-term prognoses in patients with hypertension: National Health and Nutrition Examination Study, 1999–2018. Front Cardiovasc Med. 2023;10:1099427. 36937901 10.3389/fcvm.2023.1099427 PMC10017977

[6] Guven DC, Sahin TK, Erul E, Kilickap S, Gambichler T, Aksoy S. The association between the pan-Immune-Inflammation value and cancer prognosis: A systematic review and meta-analysis. Cancers (Basel). 2022;14(11):2675. 35681656 10.3390/cancers14112675 PMC9179577

[7] Ligorio F, Fucà G, Zattarin E, Lobefaro R, Zambelli L, Leporati R, et al. The Pan-Immune-Inflammation-Value Predicts the Survival of Patients With Human Epidermal Growth Factor Receptor 2 (HER2)-Positive Advanced Breast Cancer Treated With First-Line Taxane-Trastuzumab-Pertuzumab. Cancers. 2021;13(8):1964. 33921727 10.3390/cancers13081964 PMC8073809

[8] Corti F, Lonardi S, Intini R, Salati M, Fenocchio E, Belli C, et al. The Pan-Immune-Inflammation Value in Microsatellite Instability-High Metastatic Colorectal Cancer Patients Treated With Immune Checkpoint Inhibitors. Eur J Cancer. 2021;150:155–67. 33901794 10.1016/j.ejca.2021.03.043

[9] Fucà G, Guarini V, Antoniotti C, Morano F, Moretto R, Corallo S, et al. The Pan-Immune-Inflammation Value Is a New Prognostic Biomarker in Metastatic Colorectal Cancer: Results From a Pooled-Analysis of the Valentino and TRIBE First-Line Trials. Br J Cancer. 2020;123(3):403–9. 32424148 10.1038/s41416-020-0894-7 PMC7403416

[10] von Elm E, Altman DG, Egger M, Pocock SJ, Gotzsche PC, Vandenbroucke JP. The Strengthening the Reporting of Observational Studies in Epidemiology (STROBE) Statement: guidelines for reporting observational studies. Int J Surg. 2014;12(12):1495–9. 25046131 10.1016/j.ijsu.2014.07.013

[11] Zeng R, Liu F, Fang C, Yang J, Luo L, Yue P, et al. PIV and PILE Score at Baseline Predict Clinical Outcome of Anti-PD-1/PD-L1 Inhibitor Combined With Chemotherapy in Extensive-Stage Small Cell Lung Cancer Patients. Front Immunol. 2021;29(12):724443. 10.3389/fimmu.2021.724443 PMC8586214 34777341

[12] Provenzano L, Lobefaro R, Ligorio F, Zattarin E, Zambelli L, Sposetti C, et al. The pan-immune-inflammation value is associated with clinical outcomes in patients with advanced TNBC treated with first-line, platinum-based chemotherapy: an institutional retrospective analysis. Ther Adv Med Oncol. 2023;13(15):17588359231165978. 10.1177/17588359231165978 PMC10102956 37063779

[13] Lee LE, Ahn SS, Pyo JY, Song JJ, Park Y-B, Lee SW. Pan-immune-inflammation value at diagnosis independently predicts all-cause mortality in patients with antineutrophil cytoplasmic antibody-associated vasculitis. Clin Exp Rheumatol. 2021;39 Suppl 129(2):88–93. 33200738 10.55563/clinexprheumatol/m46d0v

[14] Topkan E, Selek U, Kucuk A, Pehlivan B. Low Pre-ChemoradiotherapyPan-Immune-Inflammation Value (PIV) Measures Predict Better Survival Outcomes in Locally Advanced Pancreatic Adenocarcinomas. J Inflamm Res. 2022;18(15):5413–23. 10.2147/JIR.S385328. (PMID:36158517;PMCID:PMC9499729). 10.2147/JIR.S385328 PMC9499729 36158517

[15] Kuperminc E, Heming N, Carlos M, Annane D. Corticosteroids in ARDS. J. Clin Med. 2023;12(9):3340. 10.3390/jcm12093340 PMC10179626 37176780

[16] Walsh SR, Cook EJ, Goulder F, Justin TA, Keeling NJ. Neutrophil – lymphocyte ratio as a prognostic factor in colrectal cancer. J Surg Oncol. 2005;91(3):181–4. 16118772 10.1002/jso.20329

[17] Vuillaume LA, Lefebvre F, Benhamed A, Schnee A, Hoffmann M, Falcao FG, et al. Lymphocyte-to-C-Reactive Protein (LCR) Ratio Is Not Accurate to Predict Severity and Mortality in Patients with COVID-19 Admitted to the ED. Int J Mol Sci. 2023;24(6):5996. 36983064 10.3390/ijms24065996 PMC10051361

[18] Girndt M, Trojanowicz B, Ulrich C. Monocytes in uremia. Toxins (Basel). 2020;12(5):340. 32455723 10.3390/toxins12050340 PMC7290468

[19] Zhang F, Li L, Wu X, Wen Y, Zhan X, Peng F, et al. Pan-immune-inflammation value is associated with poor prognosis in patients undergoing peritoneal dialysis. Renal failure. 2023;45(1):2158103. 10.1080/0886022X.2022.2158103 PMC9848369 36632816

[20] Zahorec R. Ratio of neutrophil to lymphocyte counts–rapid and simple parameter of systemic inflammation and stress in critically ill. Bratisl Lek Listy. 2001;102(1):5–14. 11723675

[21] de Jager CPC, van Wijk PTL, Mathoera RB, de Jongh-Leuvenink J, van der Poll T, Wever PC. Lymphocytopenia and neutrophil-lymphocyte count ratio predict bacteremia better than conventional infection markers in an emergency care unit. Crit Care. 2010;14(5):R192. 21034463 10.1186/cc9309 PMC3219299

[22] Murat B, Murat S, Ozgeyik M, Bilgin M. Comparison of pan-immune-inflammation value with other inflammation markers of long-term survival after ST-segment elevation myocardial infarction. Eur J Clin Invest. 2023;53(1):e13872. 36097823 10.1111/eci.13872

[23] Guven DC, Erul E, Yilmaz F, Yasar S, Yildirim HC, Ercan F, et al. The association between pan-immune-inflammation value and survival in head and neck squamous cell carcinoma. Eur Arch Otorhinolaryngol. 2023;280(5):2471–8. 36565325 10.1007/s00405-022-07804-x

[24] Lin F, Zhang L-P, Xie S-Y, Huang H-Y, Chen X-Y, Jiang T-C, et al. Pan-immune-inflammation value: a new prognostic index in operative breast cancer. Front Oncol. 2022;13(12):830138. 10.3389/fonc.2022.830138 PMC9043599 35494034

[25] Qi X, Qiao B, Song T, Huang D, Zhang H, Liu Y, et al. Clinical utility of the pan-immune-inflammation value in breast cancer patients. Front Oncol. 2023;13:1223786. 10.3389/fonc.2023.1223786. (eCollection 2023). 37711203 10.3389/fonc.2023.1223786 PMC10499041

[26] Baba Y, Nakagawa S, Toihata T, Harada K, Iwatsuki M, Hayashi H, et al. Pan-immune-inflammation Value and Prognosis in Patients With Esophageal Cancer. Ann Surg Open. 2021;3(1):e113. 10.1097/AS9.0000000000000113. (eCollection 2022 Mar). 37600089 10.1097/AS9.0000000000000113 PMC10431581

